# Monitoring and optimization of POCT devices in a multi-specialty hospital in Poland: usage trends, quality assurance, and clinical impact (2017–2024)

**DOI:** 10.3389/abp.2025.14299

**Published:** 2025-06-11

**Authors:** Agnieszka Woźniak-Kosek, Lucyna Drążek

**Affiliations:** ^1^ Department of Laboratory Diagnostics, Military Institute of Medicine - National Research Institute in Warsaw, Warsaw, Poland; ^2^ Department of Laboratory Diagnostics, Laboratory of Medical Analytics in Legionowo, Military Institute of Medicine—National Research Institute in Warsaw, Warsaw, Poland

**Keywords:** point-of-care testing (POCT), critical parameter analyzers, glucometers, laboratory management, quality management

## Abstract

Rapid access to blood laboratory test results is crucial for diagnosing and treating patients in life-threatening conditions. Oxygenation status and acid-base balance are determined on arterial blood gasometry and are vital components of modern treatment algorithms for critically ill patients, similar to capillary blood glucose level measurement. The aim of this study is to present a modern method of reporting the work of critical parameter analyzers and glucometers in a multi-speciality hospital. The material for the analysis consisted of data obtained during the supervision of POCT devices in the clinics/departments/institutes of MIM-NRI from 2017 to 2024. Analyzing the use of glucometers in MIM-NRI from 2017 to 2024, it was noted that their usage significantly increased during the COVID-19 pandemic compared to the previous period. Currently, 112 devices are continuously used in both locations. All of them are subject to international and national quality testing, as well as daily internal calibration checking at their workplaces. There are currently 13 analyzers of critical parameter in the clinics and departments of MIM-NRI. All operate within the AQURE system, which allows for monitoring the correct operation of these devices and helps in quickly identifying analytical problems and reporting in real-time about the device’s operational status. In the first half of 2024, 40,082 blood sample tests were performed on patients. Analysis of correctly performed gasometric tests conducted in clinics and laboratories shows similar values, ranging from 84.17% to 86.38%. In addition to external control, critical parameter devices must necessarily undergo daily internal laboratory control and calibration. The highest number of correctly performed calibrations, 9,239, was recorded in the Cardiac Surgery Clinic’s Intensive Therapy Unit, accounting for 29% of all correctly performed calibrations for these devices. In the analyzed period of 2024, the rate of correctly performed quality checking’s was 82.26%. The highest number of correct analyses, 636, was conducted in the Intensive Therapy Unit of the hospital in Legionowo, accounting for 10.32% of all controls performed. The demand for POCT tests in Polish hospitals is steadily growing due to their enormous potential and the time savings associated with performing tests directly at the patient’s bedside. The greatest advantage of POCT is that by providing quick access to test results, diagnosis can be accelerated, and treatment initiated more rapidly. Additionally, POCT tests conducted in emergency rooms or hospital emergency departments can help to reduce the number of unnecessary hospitalizations or costly imaging tests. Having an appropriate POCT data management system in a multi-specialty hospital and ensuring IT communication is currently indispensable for proper patient care.

## Introduction

Point-of-care testing (POCT) refers to clinical laboratory tests conducted at or near the patient’s location (Nichols et al., 2007). A wide range of POCT devices are available for diagnosing both acute and chronic diseases. These devices include, glucometers, critical parameter analyzers, and tests for glycated hemoglobin, among others. POCT devices are typically simple instruments or visually interpreted test strips or kits. They enable the measurements of various parameters, such as erythrocyte sedimentation rate (ESR) and C-reactive protein (CRP) for detecting inflammation, human chorionic gonadotropin (hCG) in urine and whole blood to confirm pregnancy, and creatinine, urea, and microalbuminuria in urine and whole blood to assessing kidney function. Additionally, POCT devices are used to evaluate lipid levels (HDL, LDL cholesterol, and triglycerides), diagnose myocardial infarction, manage heart failure using troponins and brain natriuretic peptide (BNP), and assess coagulation through prothrombin time (PT), international normalized ratio (INR), activated clotting time (ACT), and activated partial thromboplastin time (aPTT).

POCT devices are also used in patient care for drug testing in urine to detect substance abuse, in emergency case management, and for conducting blood gas analysis, measuring electrolytes, and performing both basic and comprehensive metabolic panels (Schabowski et al., 2008; Park, 2021). Additionally, rapid tests are available for detecting streptococci, HIV, infectious mononucleosis, *Helicobacter pylori*, influenza virus, and, introduced during the COVID-19 pandemic, antigen tests to detect the SARS-CoV-2 virus (Díaz-Garzón et al., 2020; Isbell et al., 2021) Over the past 15 years, the range of POCT tests has expanded and continues to evolve, particularly with the introduction of new rapid molecular tests. The global POCT market is projected to exceed USD 44.6 billion by 2025, with a compound annual growth rate (CAGR) of 9% (Report on the Point-of-Care Testing, 2020).

Historically, POCT was first implemented in intensive care units, operating rooms, and emergency departments to facilitate rapid patient treatment. POCT is now transforming the traditional healthcare model, where a doctor examines a patient, orders tests, a blood sample is drawn, the sample is sent to the laboratory. The results are then communicated to the doctor through various hospital or laboratory systems, after which clinical action is taken. In model, the turnaround time for test results depends on the distance the sample travels to the laboratory and the speed of the analyzers (Isbell et al., 2021). Delays can occur, and results may not be available for several hours or even days, depending on the type of test and the complexity of the diagnostic laboratory’s workload. POCT bridges this gap by bringing the laboratory to the patient, streamlining the testing process, and significantly reducing the time required for clinical decision-making. Currently, in addition to intensive care units and emergency departments, POCT is also utilized in doctor’s offices and clinics, enabling physicians to advise patients and adjust treatments during the same visit.

In life-threatening situations, dynamic changes in laboratory parameters pose a significant challenge. Some critical laboratory parameters require immediate diagnosis and medical intervention. In such cases, testing in a central laboratory may take too long, potentially leading to the patient’s death. It is crucial to minimize the time required to perform tests, with the turnaround time (TAT) being evaluated at the point of care.

On 25 October 2023, the Regulation of the Minister of Health, dated 23 October 2023, concerning organizational standards for laboratory tests of critical parameters performed on biological material, to enable rapid therapeutic decision-making, was published in the Official Journal of the Republic of Poland. This regulation outlines the responsibilities and tasks of the laboratory diagnostician overseeing POCT testing (The Polish Regulation Act, 2023). It is the latest executive act issued under the Laboratory Medicine Act of 15 September 2022 (The Polish Legal Act, 2022). The legal regulations introduced in the field of POCT clarify aspects related to performing critical parameter tests, known as the “organizational standards for POCT.” These standards provide a list of basic laboratory tests performed within the framework of POCT, the organization of this process, and the identification of medical personnel authorized to collect samples and perform tests (Schabowski et al., 2008; Park, 2021).

## Materials and methods

The material for the analysis consisted of data obtained during the supervision of POCT devices in the clinics/departments/institutes of MIM-NRI from 2017 to 2024.

This paper will present data regarding the supervision of the most commonly used devices in the field of POCT. These include glucometers and analyzers for critical parameters. To comply with the requirements outlined in legal regulations, a Medical Diagnostic Laboratory should perform a comparative analysis of selected glucometers available on the market to determine the most optimal device for use in a particular hospital.

## Results

Authors presents an evaluation of three glucometers currently available for this purpose. The evaluation will focus on the glucometer presently used in the hospital, the Multi Sure GK by Salus International (Model 1), the StatStrip by NovaNet by CORMAY SA. (Model 2), and the Accu-Chek Inform II by Roche Diagnostics Polska Sp. z o. o. (Model 3) (see Table 1).

**TABLE 1:** Results of glucose measurements obtained from human serum for the evaluated glucometers Model 1 and 2.

Test number	Glucose measurement using glucometer model. 1 (mg/dL)	Glucose measurement using glucometer model. 2 (mg/dL)
1	105	97
2	133	125
3	222	206
4	139	125
5	140	143
6	118	112
7	104	106
8	109	100
9	138	126
10	112	124
11	166	148
12	93	70
13	196	215
14	135	153
15	212	202
16	216	191
17	207	198
18	112	104
19	158	181
20	204	208
21	180	170
22	147	153
23	98	89
24	121	115
25	99	107
26	196	180
27	153	143
28	192	198
29	167	141
30	166	146
31	145	131
32	166	164
33	176	175
34	115	98
35	154	137
36	155	136
37	145	135
38	179	186
39	124	121
40	224	198
41	137	103
42	122	112
43	133	149
44	128	94
45	127	147
46	154	213
47	195	201
48	134	118
$\bar{X}$	151,06	145,71

$\bar{X}$ ., mean value.

An analysis of the glucose results obtained from both glucometers, revealed that the readings from the currently used glucometer Model 1 were 3.54% higher than those from glucometer Model 2. Glucometer Model 1 underwent testing in hospital conditions before being put into use, and the results it provided met the international standard EN ISO 15197:2015. According to this standard, for glucose concentrations <100 mg/dL, 95% of the results should fall within ±15 mg/dL compared to those obtained by the reference method. For concentrations ≥100 mg/dL, 95% of the results should fall within ±15% of the reference method values. Based on these criteria, it can be concluded that the new device should yield results more closely aligned with those obtained from venous blood using the reference method on a laboratory analyzer.

Glucometer Model 2 underwent internal laboratory control at the Laboratory Diagnostics Department of MIM-NRI, using control materials provided with the device. Calibration measurements were performed daily from 5–8 December 2023, at two levels. The glucose values indicated in the control material leaflets were as follows:

• Control Level 2: Glu 95–115–135 mg/dL
• Control Level 3: Glu 250–300–350 mg/dL

The results are presented in Table 2.

**TABLE 2:** The results of control level 2 and 3 for glucose.

Result of control level 2 Lot No 0422321302 Expiry date 17.05.2025	Result of control level 3 Lot No 0423027303 Expiry date 27.07.2025
113	295
106	280
104	288
109	289

All obtained results fall within the acceptable glucose value ranges specified in the control material leaflets. As part of the repeatability testing of glucose measurements in a series, a glucose measurement was performed using Control level 3, Lot No. 0423027303. The results obtained are presented in Table 3. These results conform to the acceptable values and confirm the proper functioning of the device.

**TABLE 3:** Results of the obtained glucose values in Control level 3.

Result of the performed measurements	
Test number	Glucose values mg/dL
1	278
2	298
3	281
4	296
5	281
6	288
7	279
8	293
9	296
10	285
11	296
12	284
13	290
14	303
15	293
16	297
17	292
18	289
19	295
20	287
21	289
$\bar{X}$ .	290,00
SD	6,690433825
CV	2.3%
CV%	≤4.7%
X_nom_	300
BIAS%	−3.3%
acceptable BIAS%	5

$\bar{X}$ , mean value; SD, standard deviation; CV, coefficient of variation; CV%- acceptable imprecision; X_norm_, mean nominal value; BIAS%, total systematic error.

The precision check was performed in a single time series, the mean value $\bar{X}$ ., the standard deviation (SD) and the coefficient of variation (CV), which is a measure of the precision level of the method, were evaluated. The permissible imprecision was estimated based on normative data of the regulatory institutions and manufacturer’s guidelines.

Error of the correctness of the measurement method was determined as follows:

$$\text{BIAS}\%=\left[\frac{\bar{X}\;–\;{\text{X}}_{\text{nom.}}}{{\text{X}}_{\text{nom}}}\right].100$$

After performing a statistical analysis of the measurements, it was found that for Control Level 3, the accuracy and precision values set by the tester were met. The statistical calculations indicate that the glucometer Model 2 is functioning correctly.

Similarly, an evaluation was conducted of another glucometer, device Model 3, in comparison to the one currently in use, device Model 1. The results are presented in Table 4.

**TABLE 4:** Results of glucose measurements obtained from human serum for the evaluated glucometers Models 1 and 3.

Test number	Glucose measurement using glucometer Model 1 (mg/dL)	Glucose measurement using glucometer model 3 (mg/dL)
1	150	151
2	125	143
3	113	99
4	96	105
5	80	96
6	124	118
7	108	97
8	140	161
9	142	127
10	93	75
11	106	93
12	103	86
13	193	170
14	97	102
15	166	166
16	115	123
17	188	174
18	222	212
19	80	73
20	101	92
21	202	196
22	152	170
23	103	102
24	105	92
25	139	128
26	188	165
27	118	126
28	136	157
29	228	199
30	129	132
31	168	151
32	192	155
33	215	185
34	146	134
35	169	187
36	168	135
37	143	169
38	150	139
39	148	136
40	95	78
41	216	202
42	102	97
43	147	116
44	136	126
45	157	143
46	240	211
47	233	237
48	147	117
$\bar{X}$	146,13	138,5

$\bar{X}$ , mean value.

An analysis of the obtained glucose results from both devices, revealed that the readings from the currently used glucometer Model 1 were 5.22% higher than those obtained with glucometer Model 3. Glucometer Model 3 meets the requirements of the international standard EN ISO 15197:2015 and was calibrated using the reference hexokinase method.

This glucometer underwent internal laboratory calibration checking at the Laboratory Diagnostics Department of MIM-NRI, using control material provided with the device. On each day from February 12 to 15, 2024, calibration measurements were performed at two levels. The glucose values listed in the control material leaflets are as follows:

• Control Level 1: Glu 30–60 mg/dL
• Control Level 2: Glu 261–353 mg/dL

The results are presented in Table 5.

**TABLE 5:** The results of control level 1 and 3 for glucose.

Result of control level 1 Lot No 30103508 Expiry date 14.04.2025	Result of control level 2 Lot No 30103508 Expiry date 18.04.2025
44	295
44	302
44	304
44	303

All obtained results fall within the acceptable glucose value ranges provided in the control material leaflets.

On 15 February 2024, at the Laboratory Diagnostics Department of MIM-NRI, as part of the repeatability testing for glucose measurements in a series, glucose measurements were performed using Control level 1, Lot No. 30103508, and Control level 2, Lot No. 30103508. The obtained results are presented in Table 6. These results conform to the acceptable values and confirm the proper functioning of the device.

**TABLE 6:** Glucose results for Control level 1 and Control level 2.

Result of the performed measurements		
Test number	Glucose values mg/dL control level 1	Glucose values mg/dL control level 2
1	44	309
2	44	301
3	45	300
4	44	303
5	45	304
6	44	303
7	44	301
8	45	298
9	44	305
10	43	306
11	44	304
12	44	306
13	44	303
14	44	307
15	44	300
16	44	300
17	44	304
18	44	302
19	44	304
20	44	301
21	45	303
$\bar{X}$	44,1	303,05
SD	052,597	2,609,072
CV	1.2%	0.9%
CV%	≤4.7%	≤4.7%
X_norm_	45	300
BIAS%	−2.0%	1.0%
acceptable BIAS%	5	5

$\bar{X}$ , mean value; SD, standard deviation; CV, coefficient of variation; CV%- acceptable imprecision; **X**_**norm.-**_ mean nominal value; BIAS%, total systematic error.

After conducting a statistical analysis of the measurements, it was found that the established accuracy and precision values were met for the controls at both levels. The statistical calculations confirm the proper functioning of Device Model 3.

Glucometer Model 3, like Glucometer Model 2, was also positively assessed by the nursing staff based on its functionality and ease of use during the testing process.

The process-oriented approach to evaluating glucometers available on the market, as described above, enables the selection of the most optimal models to meet the needs of the hospital—in this case, a multi-specialty hospital with approximately 1,000 beds.

Analyzing the usage of glucometers at MIM- NRI from the second half of 2017 to the first half of 2024, it is evident that their use significantly increased during the years 2020–2023 compared to previous years. The usage of glucometers rose in the second half of 2020, with a notable increase in the second half of 2021, peaking in the first half of 2022. This increased use of glucometers in the MIM- NRI clinics, departments and units coincided with the COVID-19 pandemic. During this period, the Modular Hospital of MIM- NRI was opened, six glucometers. In the first half of 2023, the elevated usage of glucometers in the clinics, departments, and units of MIM- NRI remained noticeable.

**FIGURE 1:**
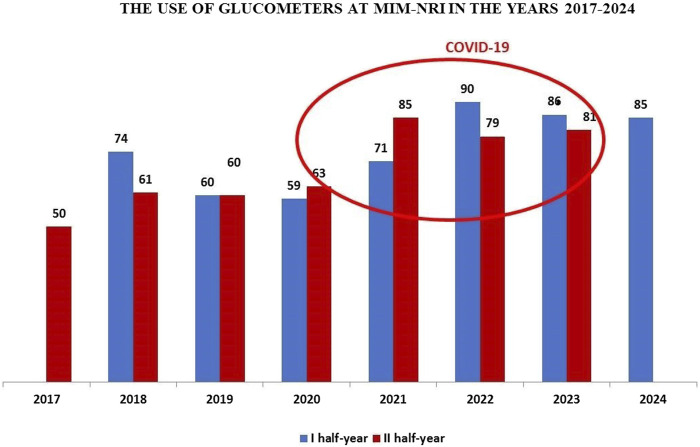
Dynamics of glucometer usage at the Military Institute of Medicine – National Research Institute (MIM- NRI).

On 1 July 2023, the state of epidemic threat was lifted across the Republic of Poland, which had been in place since May 2022. After the Labquality control of the glucometers in the first half of 2024, it was observed that the end of the pandemic did not significantly affect the use of glucometers in the MIM-NRI clinics, departments, and units, with usage levels remaining similar to those in 2023, as shown in Figure 1.

Glucometers undergo systematic international external quality control conducted by the Labquality (Finland). In May 2024, a total of 85 glucometers were subjected to Labquality’s calibration testing. The statistical evaluation of the submitted results revealed one error that exceeded the acceptable range.

For glucose, the permissible error of ±10% was exceeded by 3.04%. This exceedance of the allowable error in the first half of 2024 did not affect glucose measurements in patient samples. During the Labquality control in May, the permissible range for glucose measurements was between 58.9 and 72.1 mg/dL. The result that did not meet the external quality control requirements, obtained using this glucometer, exceeded the upper range by 2 mg/dL (a value of 74.1 mg/dL). The glucose value obtained in the control is within the reference range for glucose, which is 70–99 mg/dL. Therefore, the slightly higher glucose result on this device did not have any clinical significance.

Internal quality control performed in May and June for this glucometer, following the frequency recommended in the internal instruction “Performing Internal Quality Control of Glucose Measurements Using the Glucometer,” i.e., once a week on at least two levels of control fluids, remained within the glucose concentration ranges specified on the test strip packaging.

The described glucometer had been in use for at least 1 year and had also undergone control in 2023. The Labquality control carried out in May 2023 did not reveal any erroneous results.

In the first half of 2024, all glucometers used in the clinics and departments were subjected to external quality control conducted by Labquality.

Analyzing the results of external quality control conducted on the glucometers in constant use in the clinics, departments, and units of MIM- NRI from the second half of 2017 to the first half of 2024, it should be noted that only in the second half of 2019 were there two erroneous results exceeding the acceptable range. In the second half of 2020, errors were found in 15 glucometers, in the second half of 2023, errors were found in 5 glucometers, and one error occurred in the first half of 2024. In all other years, all results were within the permissible error range of ±10%. These results are connect on the level of 97.71%.

Example Method for Evaluating the Performance of Critical Parameter Analyzers. In the departments and clinics, there are 11 critical parameter analyzers, all of which continuously operational. These devices include the ABL 800 FLEX and ABL 90 FLEX PLUS models by Radiometer and all are integrated into the AQURE system.

**FIGURE 2:**
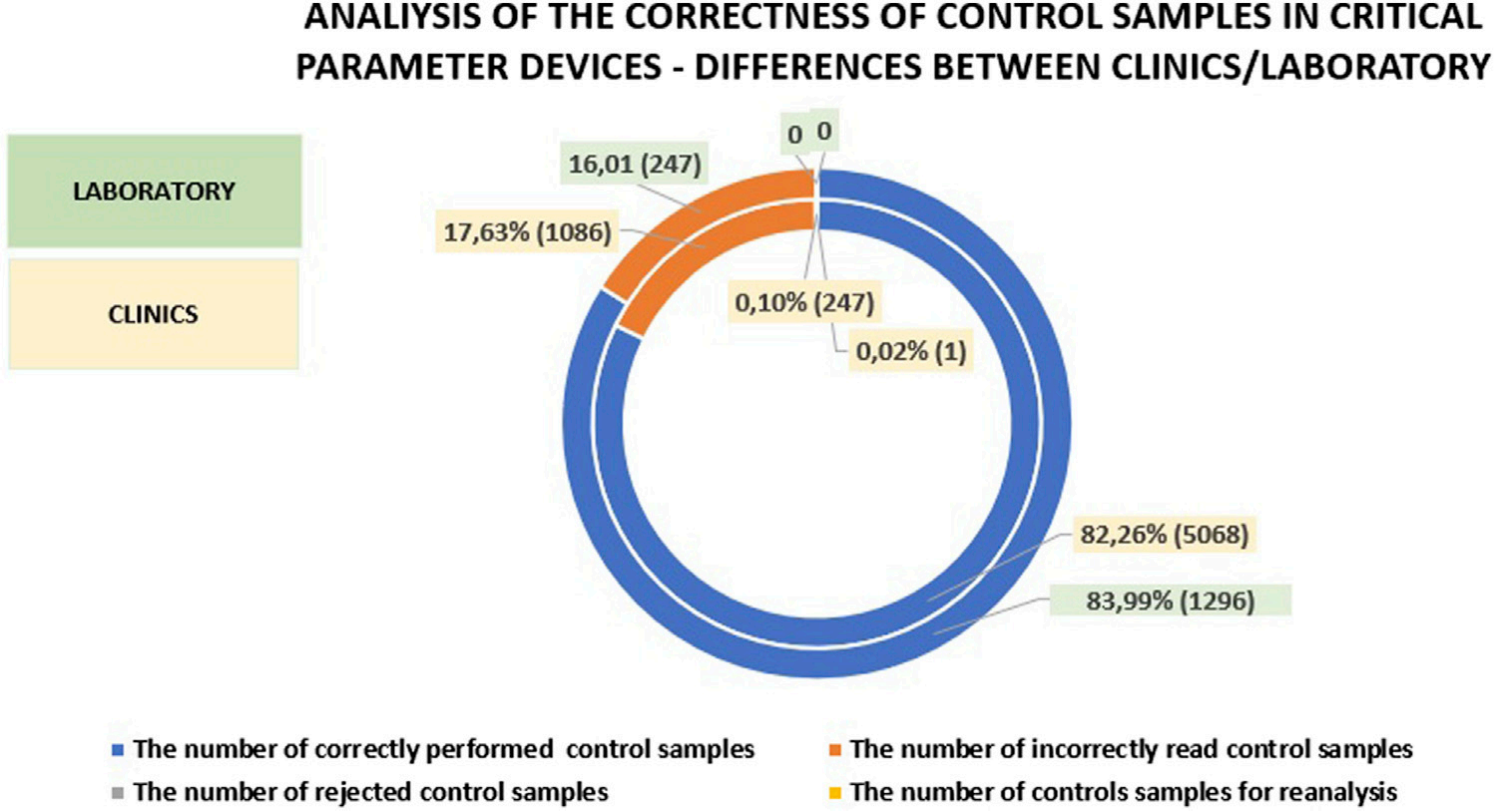
Number of tests performed using critical parameter analyzers in the first half of 2024, categorized by sample types.

The AQURE system is a software platform designed to manage analytical devices. It enables the monitoring and control events related to the operation of these devices, and facilitating prompt resolution of issues. The system continuously tracks the status of the devices, ensuring compliance with legal requirements. Figure 1 presents data collected from AQURE system for the first half of 2024. The data includes the number of patient samples processed, calibrations, quality control tests, and the quality of patient samples, categorized by status and sample collection location (see Figure 2).

An analysis of the use of critical parameter analyzers at MIN-NRI from 2017 to 2024 shows a significant increase starting in 2020, coinciding with the onset of the COVID-19 pandemic. During the hospitalization of patients infected with the SARS-CoV-2 virus, two additional critical parameter analyzers were installed at the Infectious Diseases and Allergy Clinic and the Modular Hospital at MIM-NRI.

Furthermore, we present comparative analyses of all measurements performed using critical parameter analyzers operating within the AQURE system in hospital departments, compared to those performed in the central laboratory.

**FIGURE 3:**
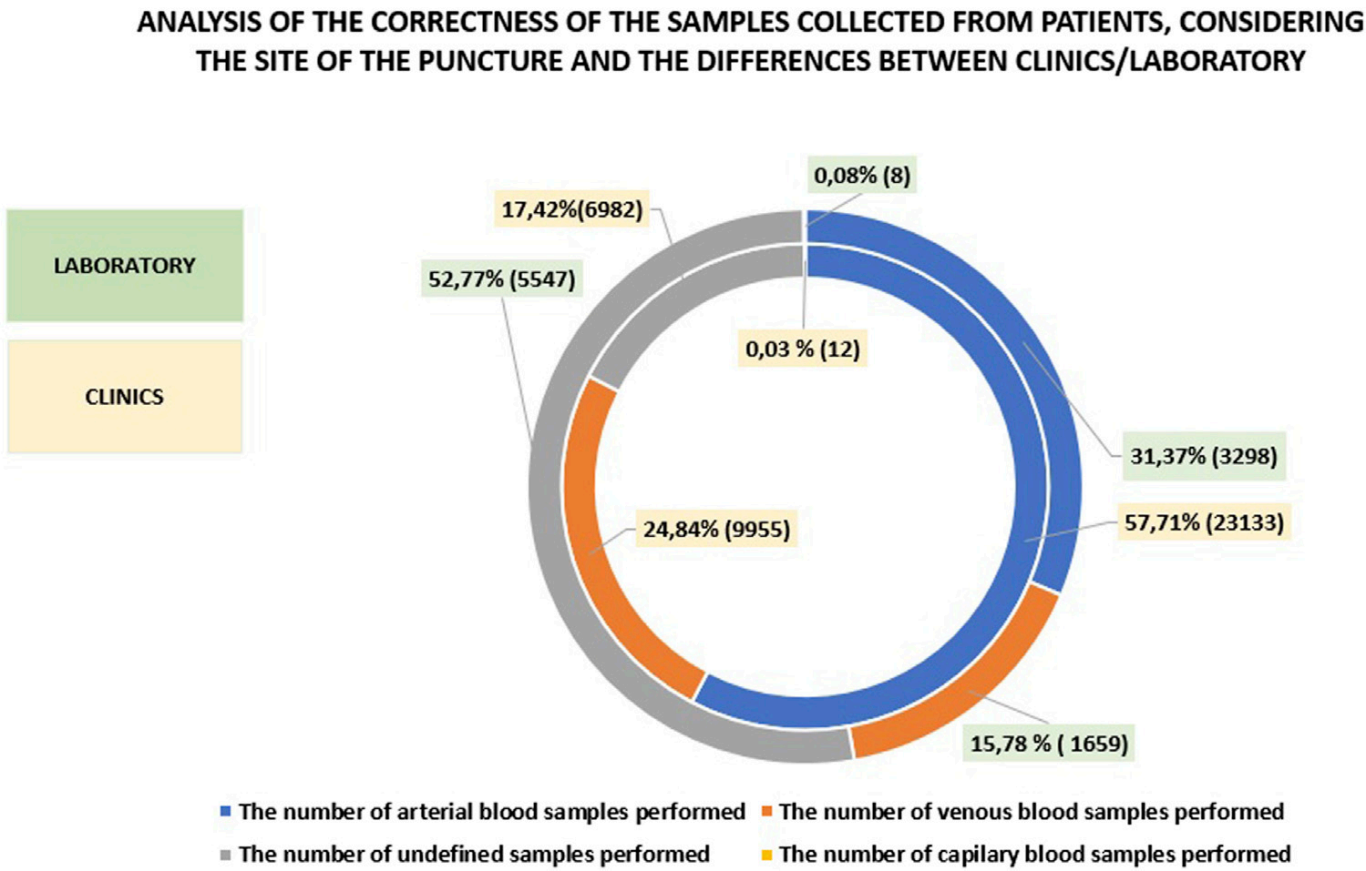
An analysis of patient sample testing compared to the percentage of controls and calibrations performed in clinics *versus* those conducted in the central laboratory.

When comparing the number of patient tests, calibrations, and controls performed on analyzers in the clinics and departments *versus* the laboratory, it is evident that fewer patient samples were processed in the laboratory (by 3.49%) and fewer controls were performed (by 0.8%). The number of calibrations performed in the central laboratory was higher by 4.29% compared to the clinics/hospital departments. This underscores the importance of POCT (Point-of-Care Testing) and supports the notion that these tests should be performed less frequently in the laboratory. Additionally, it suggests that laboratory work, due to stricter standards, necessitates more frequent calibrations, while the continuous readiness of devices in the clinics may explain the lower number of controls performed. Figure 3 presents a comparison of patient sample testing, calibrations, and controls between hospital departments and the central laboratory.

**FIGURE 4:**
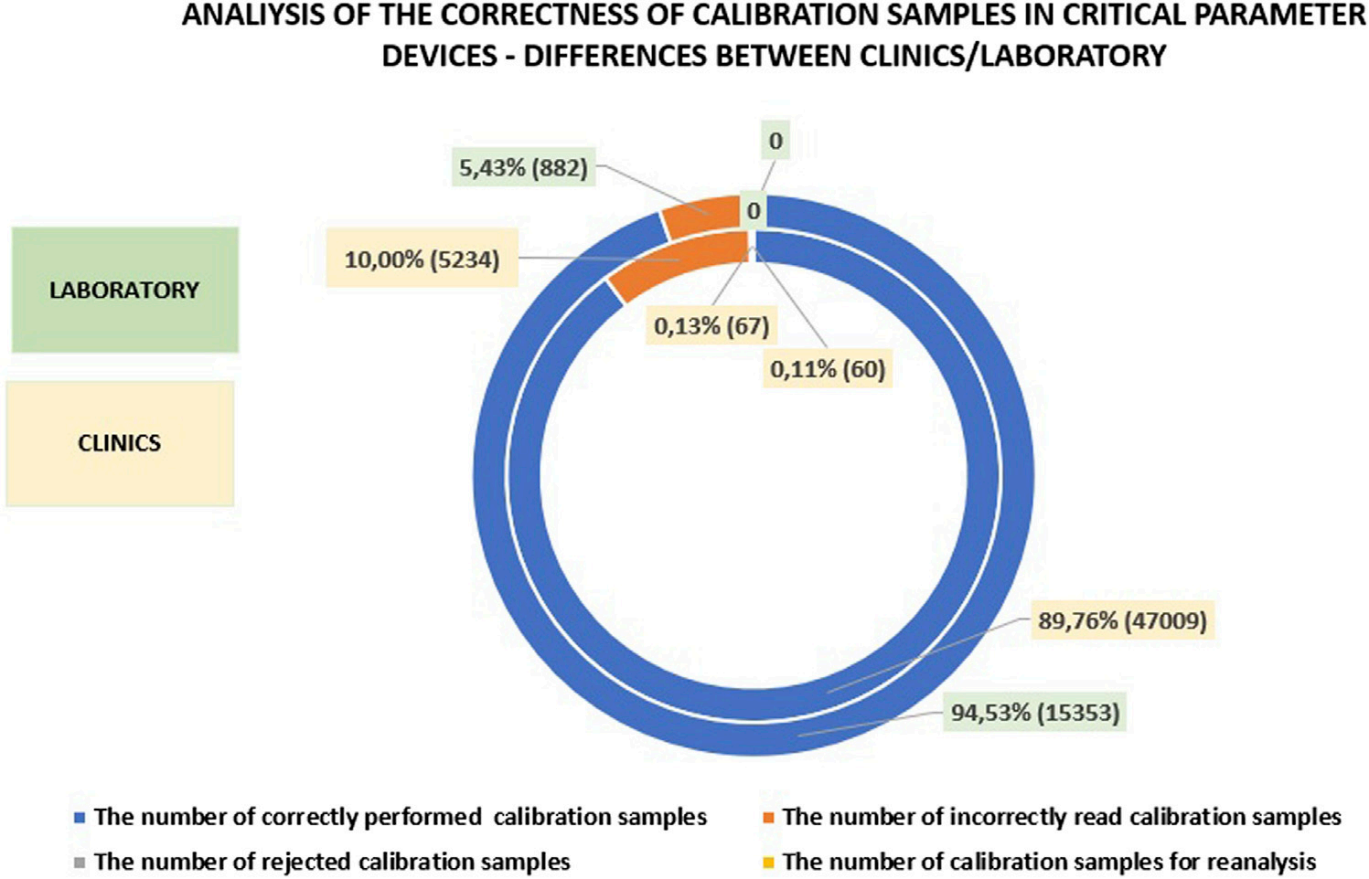
Analysis of patient samples, showing the percentage of their status in the clinics compared to those in the laboratory.

A comparison of the number of correctly performed tests on patient samples in the clinics and the laboratory reveals that the laboratory results were only 2.21% higher. The percentage of rejected, incorrectly read, or reanalyzed samples was also comparable with the laboratory showing 13.62%, while in the clinics, this figure was 15.83%. A detailed analysis is provided in Figure 3. The data clearly indicate that introducing a wide range of POCT (Point-of-Care Testing) in the hospital can yield results comparable to those obtained in the laboratory. This is made possible by the work of the POCT coordinator, a laboratory specialists who effectively supervises and supports the medical staff in this area (See Figure 4).

**FIGURE 5:**
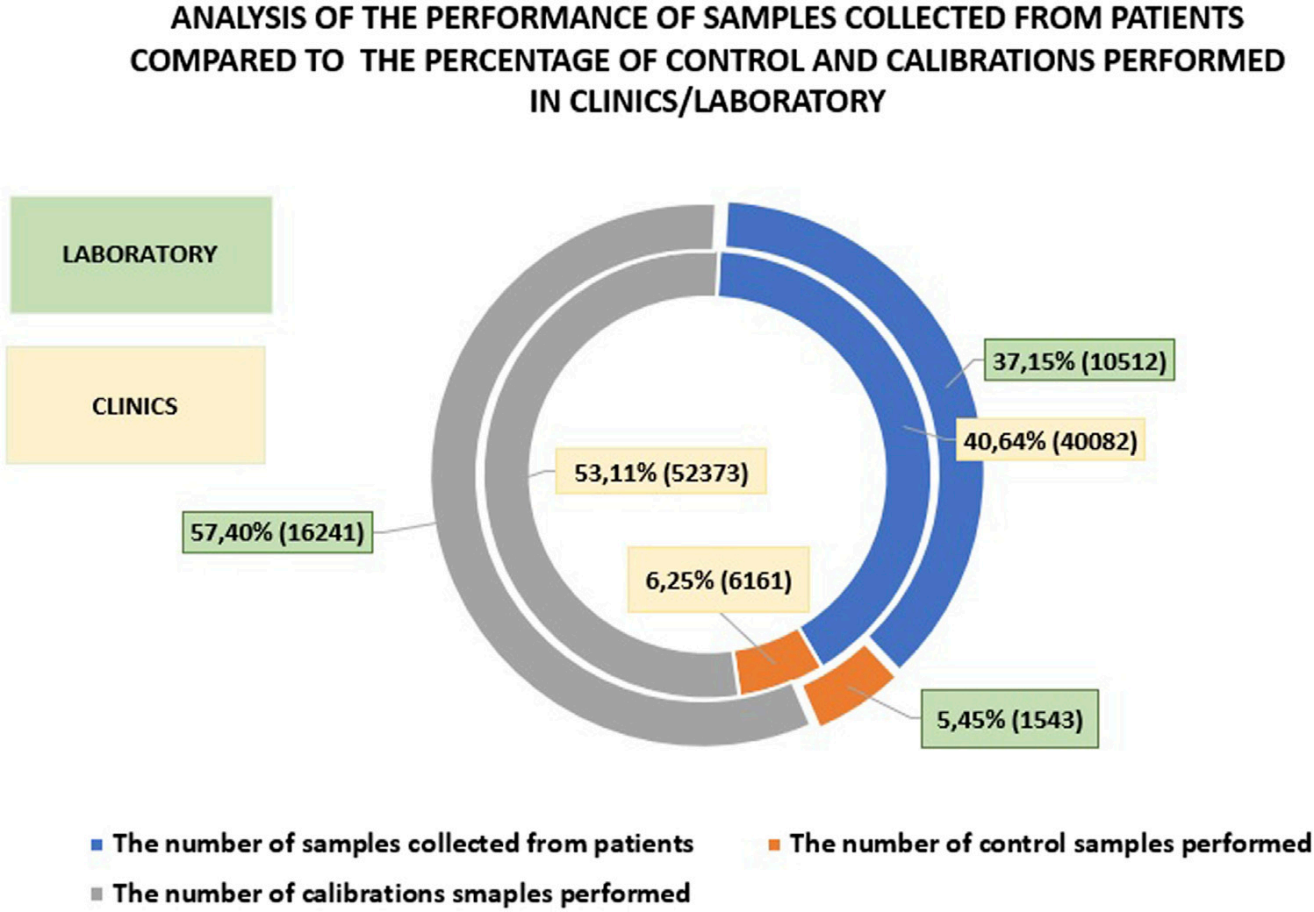
Correctness of blood collection from patients for testing.

The differences observed between the laboratory and the clinics/departments at MIM-NRI regarding the accuracy of patient sample collection, including the site of venipuncture, are presented in Figure 5. In the clinics and departments, the majority of the samples were arterial blood samples, accounting for approximately 58%, while venous blood samples represented around 25%. In contrast, the laboratory categorized the majority of samples (approximately 53%) as “undefined,” with only 31.37% being arterial blood samples and 15.78% venous blood samples. Capillary blood samples made up less than 1% in both the clinics and the laboratory. The issue of undefined samples, which comprised just over 70%, stems from. a pre-analytical error, where the sample collector fails to specify the type of material for acid-base balance analysis (e.g., not indicating whether the sample is arterial, venous, or capillary prior to analysis). As a result, the remaining samples are classified as undefined. The issue can be addressed though additional training for operators on proper handing of critical parameter analyzer.

**FIGURE 6:**
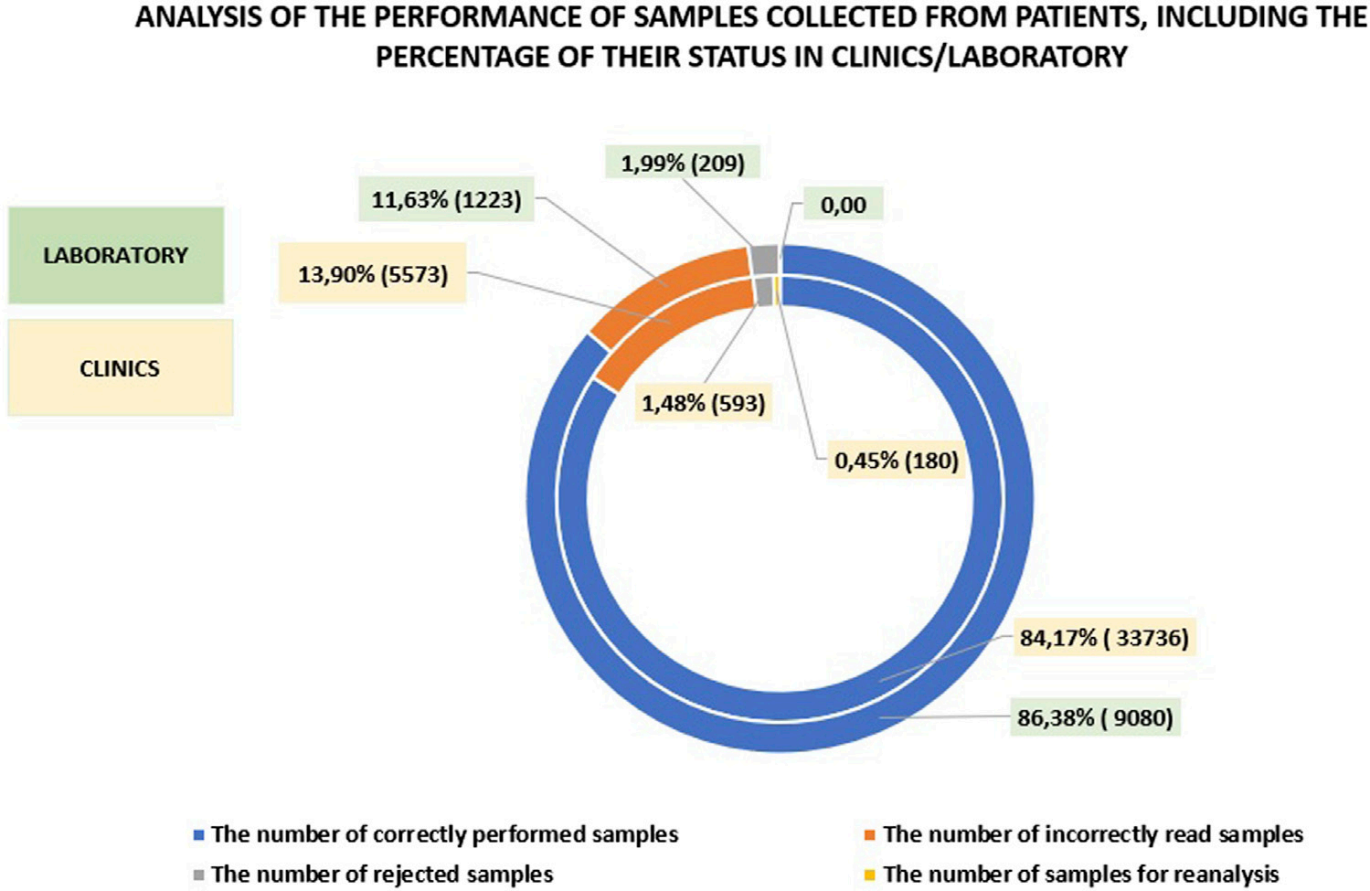
Analysis of calibration accuracy in the clinics and departments compared to the laboratory.

In the laboratory, approximately 95% of calibrations are performed correctly, which is slightly higher than in the clinics and departments. The difference in the number of incorrectly read calibrations between the laboratory and the clinics is about 5%. The minor differences in calibration accuracy can likely be attributed to the well-trained personnel operating the analyzers in both settings. The presence of skilled personnel ensures that calibration procedures are conducted with a high degree of precision, regardless of whether they are performed in the laboratory or in the clinics. Detailed data on this subject is presented in Figure 6.

In the clinics and departments, the number of correctly performed controls is comparable to that in the laboratory. However, the difference in incorrectly read calibrations is approximately 2% between the laboratory and the clinics, with significantly fewer errors occurring in the laboratory.

**FIGURE 7:**
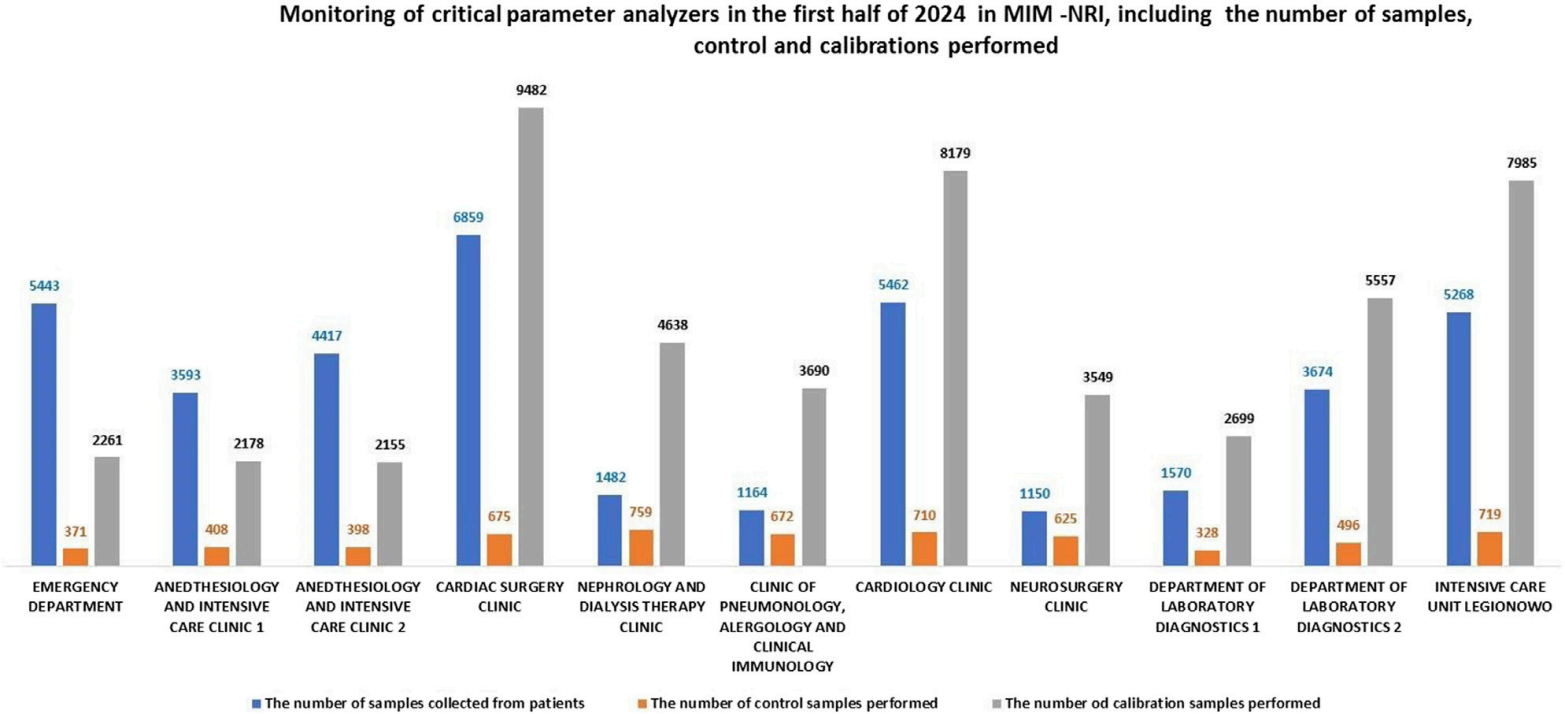
Analysis of control accuracy in the clinics and departments compared to the laboratory.

A comparative analysis suggests that continuous training of personnel in the operation of critical parameter analyzers plays a key role in reducing error. Proper training in the basic handling of analyzer can, in particular, decrease the occurrence of incorrectly performed patient sample tests, as well as the frequency of inaccurate calibrations and controls. Detailed data reading this issue are presented in Figure 7.

Critical parameter analyzers undergo regular international external quality calibration tests conducted by the Labquality and the Central Laboratory of Quality Assessment in Laboratory Diagnostics (COBJwDL) in Łódź. The results of these checking’s are transmitted electronically, and statistical reports are provided either by mail or electronically. This process enables the assessment of systematic errors in measurement procedures.

In the first half of 2024, two external quality controls assessment were carried on: one by Labquality in April and once by COBJwDL as part of the “ZIMA” program for the critical parameter analyzers in use at MIM-NRI.

• Labquality Control (April 2024): In this control, 2.81% of the results exceeded the allowable error (8 out of 285 results were incorrect).
• COBJwDL Control (ZIMA Program): During the “ZIMA” program control, 7 out of 300 results were incorrect.

In the first half of 2024, as well as in previous reporting periods, any instances where the allowable errors limits were exceeded did not impact the critical parameter measurements of patients samples. Internal quality control, performed automatically on daily on two levels by the analyzers on daily basis, consistently remained within the range defined by the manufacturer, Radiometer. Furthermore, on the days of the external control by Labquality, was conducted critical parameter measurements were also carried out using control samples from the independent company (Bio-Rad). All results were within the acceptable range.

Similarly, during the COBJwDL control under the “ZIMA”: program, 7 incorrect results were recorded out of 300 tests. However, as in previous periods, these discrepancies did not affect the critical parameter tests performed on patient samples. The daily internal quality control, conducted automatically on two levels by the analyzers, remained within the specifications defined by Radiometer, the manufacturer of the critical parameter analyzers. Furthermore, during the external control by COBJwDL, additional testing was carried out using control samples from Bio-Rad, with all results falling within the acceptable range.

## Discussion

Currently, the POCT (Point-of-Care Testing) trend is expanding beyond traditional setting such as emergency departments, hospitals, and clinics. It is increasing being utilized by medical personnel at various levels, including doctors, nurses, paramedics, and laboratory specs. POCT is also employed at large – scale events such as sports competitions, concerts, and festivals. Additionally, it is present in medical transport vehicles, including helicopters, ambulances, and is even available on cruise ships, trains, and airlines. Remarkably, a blood gas analysis device was even taken to the summit of Mount Everest (Nicholas, 2021). POCT is also being implemented in field hospitals, disaster response operations, and medical assistance in remote regions of developing countries. However, in such extreme locations, it is crucial to address the quality management of test results to ensure their accuracy and reliability (Elrobaa et al., 2024; Alter, 2021).

The recent COVID-19 pandemic has accelerated the adoption of telemedicine and remote healthcare services. Patient portals have emerged as a key tool for facilitating remote communication between doctors and patients. POCT plays a central role in these evolving healthcare models. Home testing devices enable patients to upload results directly to their medical records, allowing physicians to review them remotely. Additionally, self-testing such as PT/INR monitors, glucometers and continuous monitoring tools such as continuous glucose monitors, blood pressure cuffs, pulse oximeters, and digital scales-are increasingly being used. Wearable devices and health apps on smartwatches can track temperature and pulse, alerting users to potential sings of fever.

As a result, physicians now have unprecedented access to real-time health data, eliminating the need to wait for laboratory results (Darian et al., 2014; Florkowski et al., 2017).

However, for these advancements to be effective, it is essential to have oversight from laboratory diagnosticians who work in laboratories on a daily basis. These professionals are responsible for ensuring the proper functioning of equipment and the accuracy of tests performed under the POCT model, as demonstrated by the data presented in this article (Nichols, 2021; Woźniak-Kosek et al., 2024).

## Data Availability

I hereby declare that original data used in the preparation of this article were generated in the medical diagnostic laboratory of the Department of Laboratory Diagnostics at the Military Institute of Medicine in Warsaw. Requests to access the datasets should be directed to AW-K, kaj12@poczta.fm.
